# Meningitis and Climate in West Africa

**DOI:** 10.1371/journal.pmed.0020024

**Published:** 2005-01-25

Many different things combine to cause epidemics of disease. Among these factors are the characteristics of the infecting organism, the resistance of the host, and, as is increasingly realized, climatic conditions.

El Niño, the best known climatic disturbance, is caused by a warming of the Pacific Ocean, which then affects the climate globally. Previous work has suggested that this recurring phenomenon can have a profound effect on the incidence of many diseases, including dengue, malaria, and diarrheal diseases.

In a paper in this month's *PLoS Medicine*, Sultan and colleagues from a climate research institute and an infectious diseases center in France looked at the relation between climate and meningitis outbreaks in Mali in West Africa, a region that every year between February and May sees devastating epidemics of meningococcal meningitis affecting up to 200,000 people. The most important recurring climatic event in this region is a dry wind, known as the Harmattan, that blows throughout the winter, causing a drop in humidity and the production of vast quantities of dust.

What the authors found was that over the years 1994–2002, the week of the onset of the yearly meningitis epidemic came at around the same time as the peak of one measure of the wind—the sixth week of the year. As Pascual and Dobson say in their Perspective article on this study, “Sultan and colleagues' study is exceptional in that it illustrates a clear relationship between an external environmental variable and the initiation of disease outbreaks.”

How do climatic changes influence disease? In some cases, such as the role of flooding in spreading a waterborne disease, the causes are perhaps obvious, but why should a dry wind affect disease incidence? Previous works have suggested that the climate can work in a number of ways, by influencing the life cycle of both disease vectors and the disease-causing organism, and, as here perhaps, by affecting the resistance of the host. Sultan and colleagues speculate that the drying effects of the wind on the mucous membranes could increase the chances of the organism getting established in the human host. Whatever the causes, one very useful feature of climate is that, once the patterns are understood, they can often be predicted.

A way of predicting these meningitis epidemics could be enormously useful. Sultan and colleagues looked at only a few years, but if these findings are confirmed over a longer time period, they could make preparing for an epidemic much more efficient.

