# Correction: Meningitis and Climate in West Africa

**DOI:** 10.1371/annotation/f829edf0-96b6-4d12-a14e-a4b727e26043

**Published:** 2012-03-05

**Authors:** Meningitis and Climate in West Africa

An image that was published alongside this article has been removed because of the image's copyright status. 

